# Relationship between dietary live microbial consumption and hypertension risk in children and adolescents: results from the NHANES 2009–2023

**DOI:** 10.1016/j.jped.2026.101630

**Published:** 2026-10-27

**Authors:** Xiaowei Li, Xiaoli Li, Yong Han, Xianmei Huang

**Affiliations:** Department of Pediatrics, Affiliated Hangzhou First People’s Hospital, School of Medicine, Westlake University, Hangzhou, Zhejiang Province, China

**Keywords:** Hypertension, Probiotics, Adolescent, Obesity, Risk

## Abstract

**Objective:**

This analysis aimed to evaluate the association between dietary live microbial consumption and hypertension risk in children and adolescents.

**Methods:**

This cross-sectional analysis comprised data on children and adolescents (aged 8–17 years) from the NHANES database 2009–2023. The association between dietary live microbial consumption and hypertension risk was analyzed using logistic regression analyses. The combined effect of dietary live microbial consumption and overweight/obesity on hypertension risk was examined using interaction analysis.

**Results:**

Of these 9342 enrolled children and adolescents, 433 had hypertension, and 8909 did not. Individuals in the high-MedHi (foods containing medium and high live microbes) consumption group (vs. low-MedHi) [OR = 0.70 (0.50–0.98), P = 0.039] were associated with lower odds of hypertension, and this association persisted after further adjustment for overweight/obesity [OR = 0.71 (0.51–0.99), P = 0.046]. In the joint effect analysis of live microbial consumption (low, high) and overweight/obesity (no, yes), individuals in the high-MedHi and overweight/obesity group [OR = 0.60 (0.39–0.92)], the low-MedHi and non-overweight/obesity group [OR = 0.29 (0.20–0.42)], and the high-MedHi and non-overweight/obesity group [OR = 0.28 (0.16–0.49)] were linked to lower odds of hypertension versus the low-MedHi and overweight/obesity group, and there was a significant trend in these joint effect terms (P_trend_ = 0.019).

**Conclusion:**

High live microbial consumption was associated with reduced odds of hypertension among children and adolescents, and live microbial intake and overweight/obesity exerted a joint effect on the odds of hypertension.

## Introduction

Hypertension constitutes a major global determinant of cardiovascular and chronic kidney disease burden, driving elevated rates of mortality and disability [1]. Childhood hypertension is a significant predictor of adult-onset hypertension and the occurrence of serious cardiovascular complications later in life [2]. Children exhibiting elevated blood pressure are at heightened risk of transitioning into adulthood with clinically persistent hypertension [3]. A prevalence of 4.00% for hypertension and 9.67% for prehypertension has been documented in the population of individuals aged 19 and below [1]. Children and adolescents with overweight or obesity exhibit a markedly elevated prevalence of hypertension compared to their normal-weight peers [1]. The important risk factors for hypertension in these individuals are high body mass index (BMI), sedentary lifestyles, high-sodium diets, and family history of hypertension [4].

The gut microbiota demonstrates a significant correlation with the progression of hypertension [5]. A compromised gut barrier, resulting from microbiota dysregulation, permits the systemic entry of pathogenic microbes and their metabolites, leading to the induction of chronic inflammation and endothelial damage, which are recognized as key contributors to the pathogenesis and clinical worsening of hypertension [5]. Diet is an important external factor affecting the gut microbiota [6]. Some safe live microbes are present in fermented foods and other types of foods (e.g., yogurt, raw fruits/vegetables) and are influenced by food type and processing level. Evidence suggests that food-derived microbial components affect the gut microbiota composition and can improve gastrointestinal health and enhance immune responses [7]. Probiotic intake may be linked to lower blood pressure in the general population [8]. Furthermore, dietary microbial consumption is significantly related to a decreased risk of obesity in children and adolescents [9] and a lower risk of mortality in adults with hypertension [10]. Multiple studies have also shown that diet and BMI interact in metabolic disorders, specifically that a healthy diet may antagonize the risk of overweight/obesity-related diseases [11]. Thus, the authors intended to evaluate the association between dietary live microbial consumption and hypertension risk in children and adolescents, as well as the combined influence of microbial consumption and BMI on hypertension risk, to provide a basis for dietary management and hypertension prevention.

## Methods

### Study design and populations

The cross-sectional study utilized data from seven cycles of the National Health and Nutrition Examination Survey (NHANES), spanning 2009 to 2023. NHANES, a nationally representative survey conducted every two years by the National Center for Health Statistics (NCHS), provides cross-sectional data on the U.S. population’s health and nutritional status (https://www.cdc.gov/nchs/nhanes/). Through interviews and physical examinations, NHANES collects demographic, dietary, physical measurement, and laboratory measurement data from participants. This analysis comprised individuals aged 8–17 years, as blood pressure measurements were only conducted in participants aged ≥ 8 years. The exclusion criteria were: (1) those without complete data on dietary intake, (2) those with missing BMI data, (3) those with missing information on smoke exposure, (4) those without physical activity data, and (5) those without the assessment of hypertension. The NCHS Institutional Review Board granted ethical approval for NHANES, requiring written informed consent from each participant.

### Outcomes

The outcome was hypertension in children and adolescents. In this analysis, participants were diagnosed with hypertension if they met any one of the criteria: (1) self-reported, (2) antihypertensive medication use, and (3) clinic-measured blood pressure exceeding diagnostic cutoffs. Hypertension diagnosis was performed based on the 2017 American Academy of Pediatrics (AAP) criteria [12]: (1) individuals aged < 13 years [blood pressure ≥ 95th percentile, or ≥ 130/80 mm Hg (whichever is lower) (based on age, sex, and height)], (2) individuals aged ≥ 13 years [blood pressure ≥ 130/80 mm Hg]. In this study, systolic and diastolic blood pressure were calculated as the average of three or four measurements taken from each participant.

### Measurement of dietary live microbial intake

The dietary intake data of the participants were derived from face-to-face interviews using a 24-hour dietary recall, as data from the first 24-hour dietary recall tend to be more accurate and complete. Energy and nutrient intake for foods and beverages in the NHANES dataset were estimated through integration with the U.S. Department of Agriculture Food Surveys Nutrient Database [13]. Moreover, live microbial counts (per gram) were quantified by four domain specialists for 9388 food codes, organized into 48 distinct subgroups in the NHANES database. The content of live microbes in food is classified into three categories: low (< 10^4^ CFU/g), medium (10^4^–10^7^ CFU/g), or high (> 10^7^ CFU/g)[13].

In this analysis, the authors used two methods to classify individuals’ dietary live microbial consumption (microbial content type and consumption amount). The first method divided participants into low-, medium-, and high-live microbial groups based on the types of microbial content in food. The low-live microbial group refers to a diet that contains only low-live microbes, the medium group refers to a diet that contains medium-live microbes but does not contain high-live microbes, and the high group refers to a diet that contains high-live microbes. The second method divided the participants into low-intake and high-intake groups according to the median total intake mass of foods containing medium and high live microbes (MedHi) in their diets [MedHi median values: 1.12 100 *g*/day (i.e., 112 g/day)]. This live microbial intake reflects estimated intake of foods assigned to live microbe categories, not measured changes in the gut microbiome.

### Data collection

Participants data, including age (< 12, ≥ 12 years), smoke exposure (no, yes), overweight/obesity (no, yes), BMI percent, gender (male, female), poverty income ratio (PIR, < 1, ≥ 1, unknown), potassium (insufficient, sufficient), dietary fiber (insufficient, sufficient), the Healthy Eating Index 2020 (HEI-2020, < 50, ≥ 50, unknown), pubertal state (no, yes, unknown), physical activity (insufficient, sufficient), race (non-Hispanic White, non-Hispanic Black, Mexican American, other Hispanic, other race), and total energy were collected. Physical activity is converted into energy expenditure (MET·min) = recommended metabolic equivalent (MET) × duration of corresponding activity (minutes), with sufficient levels defined as ≥ 180 MET-min/day (12–17 years) or ≥ 60 MET-min/day (8–11 years). Smoke exposure is evaluated based on the smoking situation of family members at home. Overweight/obesity is operationalized as BMI ≥ the 85th percentile, as defined by the Centers for Disease Control (CDC) growth charts. Potassium and dietary fiber intake are classified as sufficient or insufficient based on the dietary guideline recommendations for specific age- and sex-based groups (Supplementary Table 1).

### Statistical analysis

NHANES adopts a complex multistage probability‑sampling design. Accordingly, sampling weights (WTDR2D, WTDR2DPP), stratification (SDMVSTRA), and primary sampling units (SDMVPSU) were included in all analyses to produce nationally representative estimates. Weight WTDR2D was applied for survey cycles spanning 2009‑2016 and 2021‑2023, whereas WTDR2DPP was used for 2017‑2020. Given the pooled multi‑cycle dataset, the final combined weight was calculated as: Weight = 3.2/13.2 × WTDR2DPP + 10/13.2 × WTDR2D. For descriptive statistics, continuous variables used weighted mean and standard error (SE), and categorical variables utilized weighted numbers and percentages [n (%)]. Group comparisons in continuous variables were assessed utilizing a weighted *t*-test or analysis of variance, while categorical variables were analyzed via the Rao-Scott Chi-square test. There are no missing values in the covariates.

The association between dietary live microbial consumption and the odds of hypertension was evaluated via uni- and multivariate logistic regression analyses, and the association was summarized as odds ratio and 95% confidence interval [OR (95%CI)]. All covariates in this study, including age, gender, race, PIR, physical activity, smoke exposure, total energy, potassium, dietary fiber, HEI-2020, and pubertal state, were adjusted for in the multivariate analyses. The non-linear relationship between MedHi live microbial consumption and hypertension odds was evaluated through the restricted cubic splines (RCS) curve. The combined effect of dietary live microbial consumption and overweight/obesity on hypertension odds was examined using joint effect terms. Additive interactions were quantitatively evaluated using the relative excess risk due to interaction (RERI), the attributable proportion due to interaction (AP), and the synergy index (SI): RERI = OR_11_ – OR_10_ – OR_01_ + 1; AP = RERI / OR_11_; and SI = [OR_11_ - 1] / [(OR_10_ - 1) + (OR_01_ - 1)]. A significant additive interaction is indicated when the 95%CI for RERI and AP excludes 0, and the 95%CI for SI excludes 1. Statistical analyses were carried out utilizing R 4.5.1 software (Vienna, Austria), considering P < 0.05 as significant.

## Results

The 2009–2023 NHANES surveys comprised 12,641 individuals aged 8–17 years. Through the screening process, 3299 participants were excluded, resulting in 9342 participants for final analysis (Figure 1). Of these 9342 children and adolescents, 433 had hypertension, and 8909 did not. Participant characteristics are displayed in Table 1. The mean age was 12.52 (±0.04) years, and 5296 (60.36%) individuals were at least 12 years old. Among all participants, 4620 (49.67%) were female, and 2828 (53.27%) were non-Hispanic Whites. Overweight/obesity was identified in 4012 (40.36%) children and adolescents, with 5330 (59.64%) participants classified as non-overweight/obesity. The mean consumption of MedHi live microbial food was 0.93 (±0.03) 100 *g*/day. There were 6738 (69.76%) participants in the low-MedHi consumption group and 2604 (30.24%) in the high-MedHi consumption group. Significant differences emerged in gender, overweight/obesity, BMI percent, and MedHi consumption between the hypertension and non-hypertension groups (P < 0.05).

**Fig. 1:**
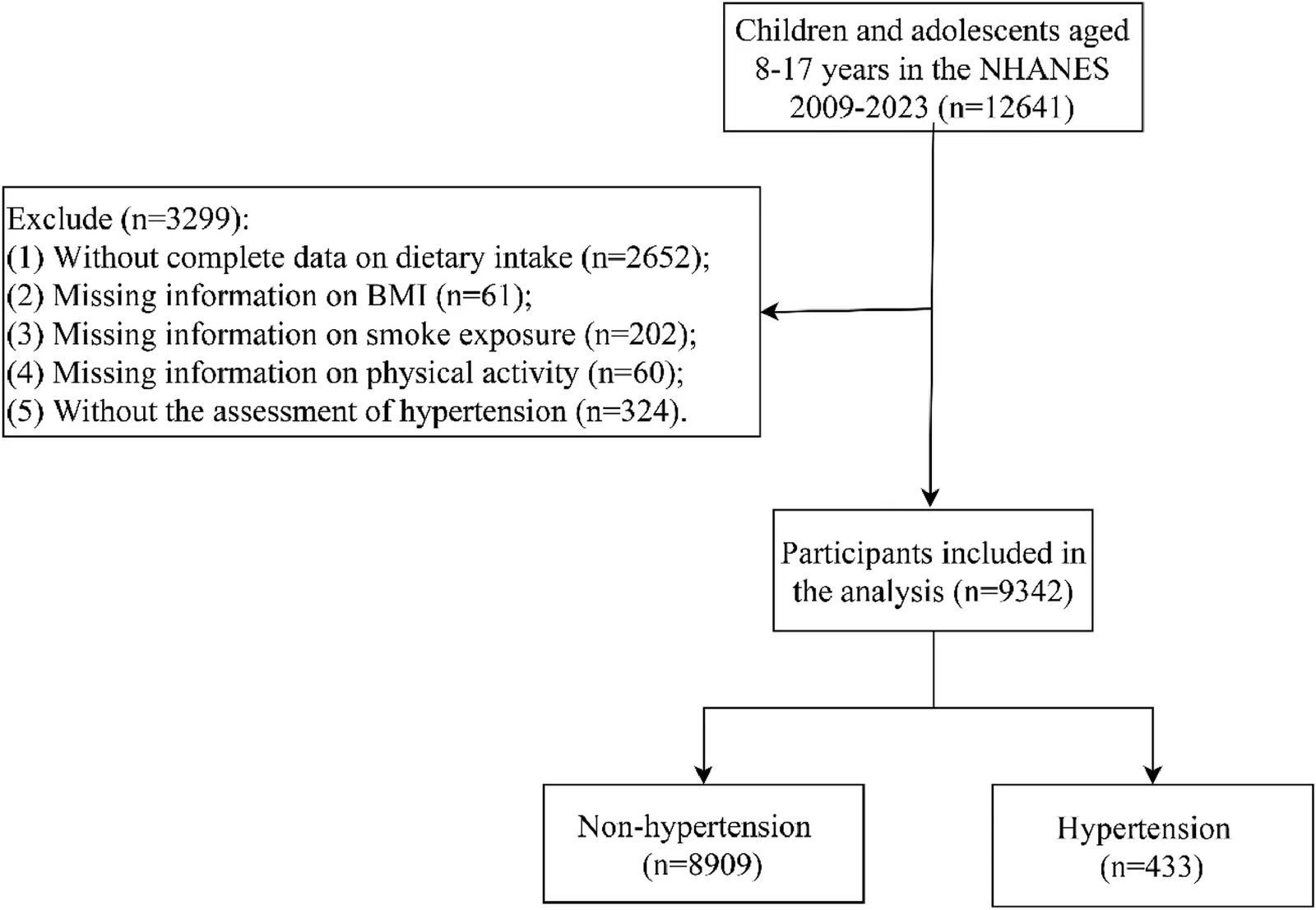
Flowchart for screening the study population. NHANES, the National Health and Nutrition Examination Survey; BMI, body mass index.

**Table 1:** Characteristics of children and adolescents.

Variables	Total (N = 9342)	Non-hypertension (N = 8909)	Hypertension (N = 433)	*P*
Age, n (%)				0.412
< 12	4046 (39.64)	3878 (39.77)	168 (36.76)	
≥ 12	5296 (60.36)	5031 (60.23)	265 (63.24)	
Gender, n (%)				0.001
Male	4722 (50.33)	4437 (49.73)	285 (63.84)	
Female	4620 (49.67)	4472 (50.27)	148 (36.16)	
Race, n (%)				0.133
Non-Hispanic White	2828 (53.27)	2714 (53.63)	114 (45.15)	
Non-Hispanic Black	2224 (13.39)	2102 (13.23)	122 (17.05)	
Mexican American	1919 (14.47)	1832 (14.29)	87 (18.38)	
Other Hispanic	1036 (8.55)	990 (8.57)	46 (8.13)	
Other race	1335 (10.32)	1271 (10.28)	64 (11.29)	
PIR, n (%)				0.079
< 1	2592 (19.94)	2454 (19.73)	138 (24.48)	
≥ 1	6168 (74.97)	5903 (75.25)	265 (68.61)	
Unknown	582 (5.09)	552 (5.01)	30 (6.90)	
Physical activity, n (%)				0.072
Insufficient	4154 (39.47)	3953 (39.16)	201 (46.45)	
Sufficient	5188 (60.53)	4956 (60.84)	232 (53.55)	
Smoke exposure, n (%)				0.213
No	8320 (90.09)	7950 (90.21)	370 (87.22)	
Yes	1022 (9.91)	959 (9.79)	63 (12.78)	
Overweight/obesity, n (%)				<0.001
No	5330 (59.64)	5189 (60.84)	141 (32.52)	
Yes	4012 (40.36)	3720 (39.16)	292 (67.48)	
BMI percent, Mean (±S.E)	68.36 (±0.58)	67.72 (±0.59)	82.79 (±1.96)	<0.001
Total energy, kcal, Mean (±S.E)	1977.83 (±15.10)	1976.26 (±15.35)	2013.26 (±52.35)	0.486
Potassium, n (%)				0.647
Insufficient	9230 (98.57)	8802 (98.56)	428 (98.94)	
Sufficient	112 (1.43)	107 (1.44)	5 (1.06)	
Dietary fiber, n (%)				0.763
Insufficient	8928 (95.66)	8504 (95.64)	424 (96.11)	
Sufficient	414 (4.34)	405 (4.36)	9 (3.89)	
HEI-2020, n (%)				0.153
< 50	4870 (46.58)	4624 (46.42)	246 (50.01)	
≥ 50	3575 (35.05)	3424 (34.98)	151 (36.68)	
Unknown	897 (18.37)	861 (18.60)	36 (13.31)	
Pubertal state, n (%)				0.367
No	1945 (22.07)	1869 (22.25)	76 (17.95)	
Yes	3527 (37.27)	3335 (37.16)	192 (39.74)	
Unknown	3870 (40.66)	3705 (40.59)	165 (42.31)	
Microbes type in foods, n (%)				0.190
Low	4136 (40.86)	3937 (40.60)	199 (46.88)	
Medium	3611 (39.63)	3460 (39.76)	151 (36.49)	
High	1595 (19.51)	1512 (19.64)	83 (16.63)	
MedHi food intake, 100 *g*/day, Mean (±S.E)	0.93 (±0.03)	0.94 (±0.03)	0.70 (±0.08)	0.003
MedHi food intake, n (%)				0.013
Low MedHi	6738 (69.76)	6407 (69.43)	331 (77.17)	
High MedHi	2604 (30.24)	2502 (30.57)	102 (22.83)	
Joint effect terms, n (%)				<0.001
Low-MedHi & overweight/obesity	2945 (28.69)	2718 (27.56)	227 (54.21)	
High-MedHi & overweight/obesity	1067 (11.67)	1002 (11.60)	65 (13.27)	
Low-MedHi & non-overweight/obesity	3793 (41.07)	3689 (41.87)	104 (22.96)	
High-MedHi & non-overweight/obesity	1537 (18.57)	1500 (18.96)	37 (9.56)	

Note: PIR, poverty income ratio; BMI, body mass index; HEI-2020, the Healthy Eating Index 2020; MedHi, foods containing medium and high live microbes.

Figure 2 lists the RCS curve of the association between MedHi food consumption and the odds of hypertension. As dietary microbial consumption increases, the odds of hypertension decrease, but no significant nonlinear association was observed (P_overall_ = 0.004, P_nonlinear_ = 0.199).

**Fig. 2:**
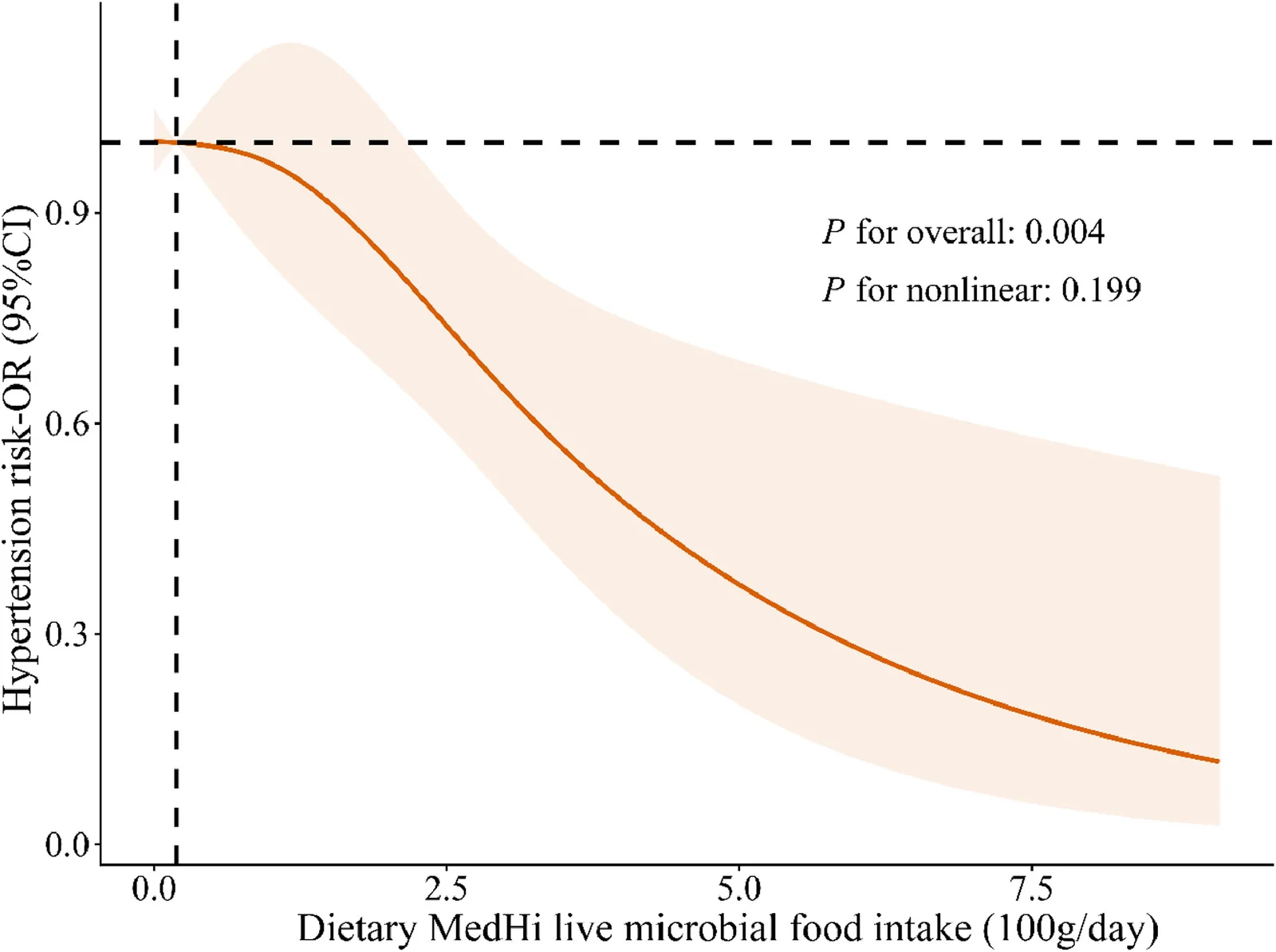
The restricted cubic splines (RCS) curve of the relationship between MedHi live microbial consumption and hypertension odds among children and adolescents. MedHi, foods containing medium and high live microbes; OR, odds ratio.

The associations between microbial food consumption and hypertension odds are presented in Table 2. When grouped according to the types of microbial content in food, the medium- (P = 0.354) and high-live microbial groups (P = 0.323) (vs. the low group) were not significantly associated with the odds of hypertension. When the analysis was based on MedHi food consumption, a 100 *g*/day increase in MedHi food intake [OR = 0.88 (0.76–1.00), P = 0.053] showed a marginally significant association with the odds of hypertension. Individuals in the high-MedHi food consumption group (vs. low-MedHi) [OR = 0.70 (0.50–0.98), P = 0.039] were associated with lower odds of hypertension, and this association persisted after further adjustment for overweight/obesity [OR = 0.71 (0.51–0.99), P = 0.046]. The association between MedHi food consumption and hypertension odds was further analyzed based on overweight/obesity subgroups (Table 3). High-MedHi food consumption was associated with lower odds of hypertension only in overweight/obese individuals [OR = 0.61 (0.39–0.95)], but not in non-overweight/obese individuals (P = 0.859).

**Table 2:** The association between live microbial food consumption and hypertension odds among children and adolescents.

Variables	Number (%)	Model 1		Model 2		Model 3	
		OR (95% CI)	*P*	OR (95% CI)	*P*	OR (95% CI)	*P*
Microbes type in foods							
Low	4136 (44.27)	Ref		Ref		Ref	
Medium	3611 (38.65)	0.79 (0.56–1.13)	0.197	0.86 (0.59–1.24)	0.409	0.84 (0.58–1.22)	0.354
High	1595 (17.07)	0.73 (0.52–1.04)	0.079	0.84 (0.58–1.21)	0.333	0.83 (0.57–1.21)	0.323
MedHi food intake	9342 (100)	0.86 (0.75–0.97)	0.017	0.87 (0.76–1.00)	0.053	0.88 (0.76–1.00)	0.053
MedHi food intake							
Low-MedHi	6738 (72.13)	Ref		Ref		Ref	
High-MedHi	2604 (27.87)	0.67 (0.49–0.92)	0.014	0.70 (0.50–0.98)	0.039	0.71 (0.51–0.99)	0.046

Note: MedHi, foods containing medium and high live microbes; OR, odds ratio; CI, confidence interval; Ref, reference.

For consecutive MedHi, OR represents the odds corresponding to a 100 *g*/day increase in MedHi.

Model 1 was a univariate logistic regression analysis.

Model 2 was a multivariate logistic regression analysis that adjusted for age, gender, race, PIR, physical activity, smoke exposure, total energy, potassium, dietary fiber, HEI-2020, and pubertal state.

Model 3 was further adjusted for overweight/obesity based on model 2.

**Table 3:** Association between MedHi live microbial consumption and hypertension odds in overweight/obesity subgroups.

Subgroups (Outcome/total)	Variables	Univariate		Multivariate	
		OR (95% CI)	*P*	OR (95% CI)	*P*
Non-overweight/obesity (N = 141/ 5330)	MedHi food intake				
	Low MedHi	Ref		Ref	
	High MedHi	0.92 (0.55–1.55)	0.751	0.95 (0.55–1.66)	0.859
Overweight/obesity (N = 292/ 4012)	MedHi food intake				
	Low MedHi	Ref		Ref	
	High MedHi	0.58 (0.38–0.89)	0.014	0.61 (0.39–0.95)	0.029

Note: MedHi, foods containing medium and high live microbes; OR, odds ratio; CI, confidence interval; Ref, reference;.

Multivariate logistic regression analysis was adjusted for age, gender, race, PIR, physical activity, smoke exposure, total energy, potassium, dietary fiber, HEI-2020, and pubertal state.

When analyzing the joint effect between MedHi food consumption (low-MedHi, high-MedHi) and overweight/obesity (no, yes) (Table 4), individuals in the high-MedHi and overweight/obesity group [OR = 0.60 (0.39–0.92)], the low-MedHi and non-overweight/obesity group [OR = 0.29 (0.20–0.42)], and the high-MedHi and non-overweight/obesity group [OR = 0.28 (0.16–0.49)] were linked to lower odds of hypertension versus the low-MedHi and overweight/obesity group, and there was a significant trend in these joint effect terms (P_trend_ = 0.019).

**Table 4:** Joint effect analysis of the association between live microbial food consumption and overweight/obesity on hypertension odds among children and adolescents.

Variables	Number (%)	Univariate		Multivariate	
		OR (95% CI)	*P*	OR (95% CI)	*P*
Joint effect terms					
Low-MedHi & overweight/obesity	2945 (31.52)	Ref		Ref	
High-MedHi & overweight/obesity	1067 (11.42)	0.58 (0.38–0.89)	0.014	0.60 (0.39–0.92)	0.020
Low-MedHi & non-overweight/obesity	3793 (40.60)	0.28 (0.20–0.40)	<0.001	0.29 (0.20–0.42)	<0.001
High-MedHi & non-overweight/obesity	1537 (16.45)	0.26 (0.15–0.43)	<0.001	0.28 (0.16–0.49)	<0.001
Trend for *P*	0.019				

Note: MedHi, foods containing medium and high live microbes; OR, odds ratio; CI, confidence interval; Ref, reference;.

Multivariate logistic regression analysis was adjusted for age, gender, race, PIR, physical activity, smoke exposure, total energy, potassium, dietary fiber, HEI-2020, and pubertal state.

Table 5 shows the results of the additive interaction between MedHi food consumption and overweight/obesity on hypertension odds. The RERI for the additive interaction was −1.33 (−2.62, −0.04), the AP was −0.65 (−1.41, 0.10), and the SI was 0.44 (0.19, 1.00), indicating that the additive interaction was not significant. However, high microbial consumption may exert an antagonistic trend on the odds of hypertension associated with overweight/obesity. Figure 3 visualizes the interaction of microbial consumption and BMI on the odds of hypertension. With the increase of MedHi microbial consumption, the slope of BMI relative to hypertension odds gradually decreases, indicating that high levels of microbial consumption may exert an antagonistic trend on the odds of BMI-related hypertension. As BMI increases, the impact of microbial consumption on hypertension also increases. Furthermore, multiplicative interaction analysis revealed no significant multiplicative interaction between MedHi microbial consumption and overweight/obesity in relation to the odds of hypertension (P for likelihood‑ratio test = 0.470) (Supplementary Table 2).

**Table 5:** Additive interaction between MedHi microbe intake and overweight/obesity on hypertension odds.

Measures	Univariate		Multivariate	
	Measure (95% CI)	*P*	Measure (95% CI)	*P*
Low MedHi & Lean	Ref		Ref	
Low MedHi & Overweight/obesity	3.59 (2.53–5.09)	<0.001	3.42 (2.39–4.89)	<0.001
High MedHi & Lean	0.92 (0.55–1.54)	0.751	0.95 (0.56–1.63)	0.855
High MedHi & Overweight/obesity	2.09 (1.32–3.31)	0.002	2.04 (1.27–3.27)	0.003
RERI	−1.42 (−2.78, −0.06)		−1.33 (−2.62, −0.04)	
AP	−0.68 (−1.46, 0.10)		−0.65 (−1.41, 0.10)	
SI	0.43 (0.19, 0.98)		0.44 (0.19, 1.00)	

Note: MedHi, foods containing medium and high live microbes; OR, odds ratio; CI, confidence interval; Ref, reference; RERI, the relative excess risk due to interaction; AP, attributable proportion due to interaction; SI, the synergy index.

Multivariate logistic regression analysis was adjusted for age, gender, race, PIR, physical activity, smoke exposure, total energy, potassium, dietary fiber, HEI-2020, and pubertal state.

**Figure 3:**
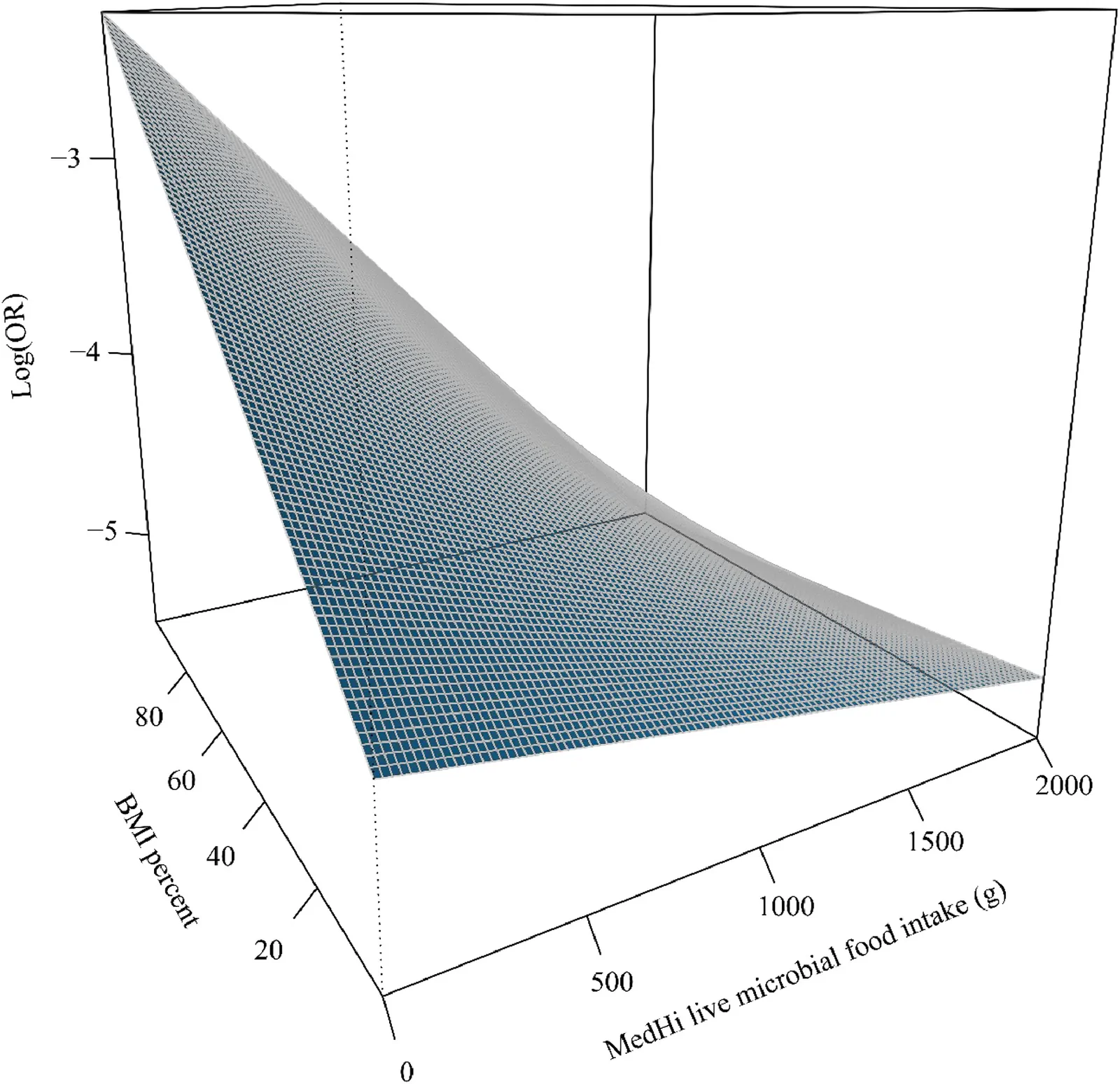
The visualization of the interaction between dietary live microbial consumption and BMI on the odds of hypertension. BMI, body mass index; MedHi, foods containing medium and high live microbes; OR, odds ratio.

## Discussion

The present study explored the association between microbial consumption and the odds of hypertension in children and adolescents. The findings indicated that high MedHi microbial consumption was associated with lower odds of hypertension among children and adolescents. Moreover, live microbial intake and overweight/obesity exhibited a joint effect on the odds of hypertension.

Hypertension prevalence increases steadily with advancing age. Childhood blood pressure status correlates closely with that in early middle age, and this status can persist from childhood into adulthood [14]. The gut microbiota has been reported to regulate blood pressure through various mechanisms, encompassing the renin-angiotensin-aldosterone, autonomic nervous, and immune systems [5]. Diet is regarded as one of the most influential modifiable factors shaping gut microbial diversity and function [15]. The present findings revealed that high MedHi microbial food consumption was associated with lower odds of hypertension. A meta-analysis also showed that probiotic intervention significantly reduced blood pressure, BMI, and glucose levels in hypertension patients [16]. In addition, these findings indicated that high microbial consumption may exert an antagonistic trend on the odds of overweight/obesity-related hypertension. Overweight/obesity is an important factor influencing hypertension. Hypertension has a prevalence of 15.3% among children with obesity, while it is 1.9% among children of normal weight [1]. Hill et al. reported that a diet rich in live microbes is linked to reduced systolic blood pressure, BMI, C-reactive protein, blood glucose, and triglyceride levels [17]. A meta-analysis demonstrated that probiotic consumption has a significant effect on reducing weight, BMI, fat mass, and insulin [18]. These studies demonstrated the reducing effect of high dietary live microbial consumption on the characteristics related to childhood obesity.

Hypertension pathogenesis in children and adolescents is multifaceted, wherein activation of the immune and sympathetic nervous systems exerts key regulatory effects [19]. Multiple critical components of the immune system are thought to participate in the pathogenic process of hypertension, such as adipocytokines, matrix metalloproteinases, tissue inhibitors of metalloproteinases, and the adaptive immune responses driven by T cells [20]. Adiponectin, a type of adipocytokine, is negatively linked to hypertension risk, even in subjects with normal BMI, and this correlation increases with age [21]. Immune system cells secrete matrix metalloproteinases and tissue inhibitors to control extracellular matrix remodeling, and these molecules mediate cardiovascular remodeling in hypertension [20]. Adolescents with hypertension exhibit changes in the distribution of adaptive immune T cells, and the distribution pattern of T cell subsets is related to left ventricular hypertrophy and aortic stiffness [22]. Some speculative mechanisms may be applicable to the influence of dietary live microbial intake on hypertension in children and adolescents. Live microbial strains are able to augment immune responses by engaging with gut-associated lymphoid tissue, inducing the synthesis of anti-inflammatory cytokines, enhancing the functional activity of regulatory T cells, and restoring the homeostatic balance between pro- and anti-inflammatory mediators [17]. Increased sympathetic nervous activity also contributes to the development of hypertension [23]. The sympathetic nervous system regulates blood pressure levels by inducing peripheral vasoconstriction, managing renal water and sodium balance, and stimulating renin release from juxtaglomerular cells [19]. Communication between the gut and brain may activate sympathetic nerves, contributing to the development of hypertension [24]. Furthermore, probiotics may reduce blood pressure through angiotensin-converting enzyme-inhibitory peptide secretion and lipopolysaccharide signaling interference, ameliorating endothelial dysfunction [5,17]. The gut microbiota may also link to the pathological process of hypertension through the brain-gut-kidney axis, inflammatory factors, and metabolites (e.g., short-chain fatty acids) [25].

This study supplements evidence on the relationship between dietary live microbial consumption and the odds of hypertension among children and adolescents. Additionally, the authors further investigated the combined effect of dietary live microbial intake and BMI on hypertension incidence. The analysis data are sourced from the nationally representative NHANES database, with all data subjected to rigorous quality control for validity assurance. Nevertheless, some limitations of this study must be acknowledged. First, NHANES dietary data originates from 24-hour dietary recall interviews, which may introduce recall bias and may not accurately quantify dietary intake. Furthermore, this only reflects recent dietary intake levels. Second, since laboratory test indicators such as blood glucose are only measured in adolescents and older subjects, the metabolic status of children and adolescents has not been included in the analysis, which may affect the results. Third, it is not possible to determine the causal connection between microbial consumption and hypertension due to the cross-sectional design of this study. Fourth, the present study population consisted of children and adolescents in the U.S. Due to differences in dietary patterns, the applicability of the study results to other populations should be further verified. Fifth, single-day dietary recall, residual confounding factors, misclassification of microbial intake, and variability in blood pressure measurements in children may all affect the accuracy of the results.

In conclusion, high dietary live microbial intake shows a weak inverse association with hypertension in children and adolescents, but findings may be confounded. Furthermore, dietary live microbial intake and overweight/obesity exerted a joint effect on the odds of hypertension. Prospective studies are needed.

## Ethics approval and consent to participate

Not applicable, because NHANES belongs to public databases; the patients involved in the database have obtained ethical approval; users can download relevant data for free for research and publish relevant articles; the present study is based on open-source data, and the Hangzhou First People’s Hospital Affiliated to Westlake University School of Medicine does not require research using publicly available data to be submitted for review to their ethics committee, so there are no ethical issues and other conflicts of interest. The NCHS Institutional Review Board granted ethical approval for NHANES, requiring written informed consent from each participant.

## Consent for publication

Not applicable, because this paper did not reveal any personal information of patients.

## Funding

This research received no specific grant from any funding agency in the public, commercial, or not-for-profit sectors.

## Authors' contributions

Xiaowei Li and Xianmei Huang conceived and designed the study.

Xiaowei Li, Xiaoli Li and Yong Han collected the data.

Xiaowei Li, Xiaoli Li and Yong Han analyzed and interpreted the data.

Xiaowei Li wrote the manuscript.

Xianmei Huang provided critical revisions that are important for the intellectual content.

All authors approved the final version of the manuscript.

## Data availability

The datasets used and/or analyzed during the current study were publicly available from the NHANES database, https://www.cdc.gov/nchs/nhanes/

## Conflicts of interest

The authors declare no conflicts of interest.

## Appendix Supplementary materials

Image, application 1
