# Supplementary material for: Relationship between dietary live microbial consumption and hypertension risk in children and adolescents: results from the NHANES 2009–2023
Source: J Pediatr (Rio J). 2026 Oct 27;103(1):101630. doi: 10.1016/j.jped.2026.101630 (PMC13733419; doi:10.1016/j.jped.2026.101630)
Supplement: Supplementary file 1 [file mmc1.docx]

**JPED-D26-00128_Supplementary Material**

**Supplementary Table 1** Age-sex groups based on dietary reference intakes and dietary guidelines recommendations.

| Macronutrients | Source of Goal | Child 1-3 | Female 4-8 | Male 4-8 | Female 9-13 | Male 9-13 | Female 14-18 | Male 14-18 |
| --- | --- | --- | --- | --- | --- | --- | --- | --- |
| Dietary Fiber, g | 14 g/1,000 kcal | 14 | 16.8 | 19.6 | 22.4 | 25.2 | 25.2 | 30.8 |
| Potassium, mg | Adequate Intake | 3,000 | 3,800 | 3,800 | 4,500 | 4,500 | 4,700 | 4,700 |

Available at: https://odphp.health.gov/sites/default/files/2019-09/2015-2020_Dietary_Guidelines.pdf

**Supplementary Table 2** Multiplicative interaction between MedHi microbe intake and overweight/obesity on hypertension risk.

| Variables | Univariate | | Multivariate | |
| --- | --- | --- | --- | --- |
|  | Likelihood‑ratio test | *P* | Likelihood‑ratio test | *P* |
| Overweight/obesity × MedHi food intake | -1695.8 |  | -1661.1 |  |
|  | -1695.7 | 0.624 | -1660.9 | 0.470 |

Note: MedHi, foods containing medium and high live microbes;

Multivariate model was adjusted for age, gender, race, PIR, physical activity, smoke exposure, total energy, potassium, dietary fiber, HEI-2020, and pubertal state.
